# The Effects of Pulsed Electrospinning Process Variables on the Size of Polymer Fibers Established with a 2^3^ Factorial Design

**DOI:** 10.3390/polym16162352

**Published:** 2024-08-20

**Authors:** Aleksandra Bartkowiak, Marcin Grzeczkowicz, Dorota Lewińska

**Affiliations:** Nalecz Institute of Biocybernetics and Biomedical Engineering Polish Academy of Sciences, Ks. Trojdena 4, 02-109 Warsaw, Poland; mgrzeczkowicz@ibib.waw.pl (M.G.); dlewinska@ibib.waw.pl (D.L.)

**Keywords:** electrospinning, pulsed voltage, polymer fiber, factorial design, bimodal structure

## Abstract

In the present study, the influence of the electrical parameters of the pulsed electrospinning process, such as the electrical voltage, the frequency of pulses, and the pulse duration, on the structure of obtained nonwovens was determined for the first time. It was found that all the parameters studied strongly influence the average diameter of the obtained fibers and that the pulsed electrospinning process carried out under specific conditions makes it possible to obtain, among other things, bimodal nonwovens. A 2^3^ factorial design was used to determine how the selected electrical parameters of the pulsed electrospinning process affect the structure of the resulting electrospun mats. It is shown, among other things, that by appropriately selecting the parameters of the electrospinning process, the thickness of fibers can be controlled, resulting in nonwovens with a desired morphology.

## 1. Introduction

Electrospinning is an efficient technique for obtaining continuous polymer fibers with a controlled geometry and with diameters ranging from 2 nm to a few micrometers. The main advantage of this method is that it is relatively simple and inexpensive to produce fibers, as the process takes place at room temperature and under atmospheric pressure. Electrospun fibers offer a high surface area-to-volume ratio, controllable porosity, and the possibility to manipulate the fiber’s composition to achieve desired properties and functions. Furthermore, the fiber scaffolds produced by this method reflect the natural extracellular matrix very well compared to conventional techniques. Hence, a staggering number of fibrous materials have been developed over the past few decades for applications including filtration, enzyme immobilization, controlled drug release systems, tissue engineering, and dressing manufacturing [1,2]. By modifying the process parameters, nonwovens with a specific morphology and fiber thickness can be obtained. In addition, the finished nonwovens can be modified by, for example, coating them with a thin polymer film, adding carbon nanotubes [3], or introducing microspheres into the structure [4], thereby giving the nonwovens increasingly attractive functional properties. It should also be noted that the only major limitation of the electrospinning method is the low efficiency of fiber production [1,2].

There is an extremely wide range of biodegradable and biocompatible natural (chitosan, collagen, gelatin, silk, and alginate) and synthetic polymers (polyvinylpyrrolidone, poly (lactic acid), polyacrylonitrile, poly (ethylene oxide), poly (acrylic acid), polyacrylamide, and poly (vinyl alcohol)), both in the form of homopolymers, copolymers, and blends. One of the most widely used synthetic polymers in electrospinning is polyvinylpyrrolidone (PVP) due to its excellent biocompatibility, remarkable solubility in water and most organic solvents, and its ability to interact with a wide range of hydrophilic and hydrophobic materials [5,6].

In the electrospinning process, the polymer is pumped into a spinning nozzle to which a strong electric field is applied. This results in the distribution of electrical charges on the surface of the polymer jet leaving the spinning nozzle. One electrode, with a positive potential, is connected to the nozzle with the polymer solution and the other, with a negative potential, to a grounded collector. When the electrical voltage increases, the semicircular surface of the polymer at the exit of the nozzle elongates to form a characteristic conical shape called a Taylor cone. A further increase in the electrical voltage, up to a critical value, causes the Coulombic forces, responsible for the repulsion of opposing charges, to overcome the surface tension forces of the polymer jet. The discharged polymer solution beam is ejected from the nozzle tip towards the collector. Meanwhile, the solvent is evaporated, and fibers are collected on the grounded collector [7,8,9].

The parameters affecting the formation of fibers during the electrospinning process and their morphology can be divided into two main groups: (1) the physicochemical properties of the polymer solution, such as viscosity, conductivity, and surface tension, which depend on the molecular weight of the polymer, the type of solvent, and the concentration of the polymer solution and (2) the parameters of the electrospinning process, such as the electrical voltage, the flow rate of the polymer solution, the distance between the spinning nozzle and the collector, and environmental parameters (the temperature, humidity, and airflow in the chamber) [1,7,10]. Understanding how each parameter affects the product obtained by electrospinning is crucial to achieving the desired result.

As is well known, the electrical voltage influences the electrical force that stretches the polymer jet into the finest fiber and thus affects the diameter of the fibers and the morphology of nonwovens. It is widely accepted that electrical voltage is one of the most important parameters determining the morphology and diameter of the fibers obtained by the electrospinning process. A direct-current voltage (DCV) is commonly used in the electrospinning process [11,12,13], while a pulsed voltage (PV) is used much less frequently [4,14,15,16]. In the second process variant, an electrical voltage is applied to the nozzle in the form of pulses of a preset frequency and pulse duration. The use of a pulsed voltage (PV) in the electrospinning process, therefore, provides an additional opportunity to control parameters such as the frequency of the pulses and the pulse duration, which can significantly affect the morphologies of the obtained nonwovens.

In one of the first papers on the use of a pulsed voltage (PV) in the electrospinning process, Li et al. [14] reported the effects of the electrical voltage, the flow rate of a polyvinylpyrrolidone (PVP) solution, the frequency and pulsed electric field duty cycle on the average diameter, and the diameter distribution of electrospun fibers. Among other things, it was found that the effects of frequency on fiber diameter was not significant. Furthermore, the use of PV allowed for thinner fibers to be obtained. In turn, Mirek et al., in their work [16], demonstrated that the type of electric voltage applied in the process influences the structure of the produced electrospun mats. The electrospinning process of polyvinylpyrrolidone (PVP) and polylactide (PLA) solutions was carried out using a direct-current voltage (DCV) and a pulsed voltage (PV) of selected frequencies (20, 50, and 100 Hz) and a pulse duration of 5 ms. The parameter that had the strongest effect on the structure of the fiber mats was the frequency of the pulses. In addition, the authors found that the use of a pulsed voltage (PV) provided, among other things, a better stability of the electrospinning process and allowed for a greater variation in the structure of the resulting electrospun mats.

For a very long time, it was thought that the ideal nonwoven fabric consisted of smooth and homogeneous fibers with a relatively narrow distribution of average fiber diameters. In recent years, it has been proven that the production of nonwoven fabrics with bimodal and multimodal structures, as well as those containing spheroidal elements in the structure (e.g., bead-on-string nonwoven fabrics), open up new possibilities for biomedical engineering applications, among others [16,17,18,19,20,21,22]. Hence, it seems necessary to determine how changing the electrical parameters of the pulsed electrospinning process will affect the morphology of the resulting nonwovens.

In earlier work, it was found, among other things, that the thickness of smooth fibers depends mainly on the electrical voltage (to a much lesser extent it depends on the viscosity and flow rate of the solution used for electrospinning), while the thickness of beaded fibers depends on the viscosity of the solution as well as the electrical voltage [23]. Additionally, the morphological and mechanical properties of the obtained nonwovens show a clear correlation with the applied voltage [24]. Furthermore, the morphology of the produced nonwovens is influenced by both the waveform (square, sine, and triangle) and the frequency of the applied alternating current (AC) high-voltage signal [25]. As a result, Sivan et al. [25] obtained nonwovens containing smooth fibers and those with beads, spindles, and/or spiral fibers in their structure. Furthermore, the authors also demonstrated that higher frequencies favor the formation of thicker fibers.

The aim of this study was to determine the influence of all electrical parameters, such as the electrical voltage (*U*), the frequency of the pulses (*f*), and the pulse duration (*τ*), on the pulsed electrospinning process, the structure of the resulting nonwovens, and the thickness of the fibers. A polyvinylpyrrolidone (PVP) solution was electrospun using a pulse voltage (PV) with a set frequency in the range of 10–100 Hz and a pulse duration in the range of 1–9 ms. A 2^3^ full factorial design was used to determine how the selected electrical parameters of the electrospinning process affect the structure of the resulting electrospun mats. A factorial design is widely used to study the effects of experimental factors and the interactions between these factors, that is, how the effect of one factor changes with the level of the other factors in the response. The advantages of a factorial design are its relatively low cost, the much smaller number of experiments required, and the increased ability to assess interactions between variables [26,27].

Only a few papers have used a factor analysis to evaluate the properties of the solution used for electrospinning and the effects of the process parameters on the morphology of the produced nonwovens [23,28,29,30,31]. However, the authors’ conclusions are clear: a factor analysis is a useful tool for designing fiber mats with the desired structure and properties to meet the requirements of various applications, i.e., drug delivery, dressings, or tissue engineering. The 2^3^ full factorial design was previously successfully applied by Korycka et al. [23], among others, to determine the relationship between factors, i.e., the flow rate of a polyvinylpyrrolidone (PVP) solution, the solution viscosity, and the electrical voltage, and the diameter of homogeneous and bead-on-string fibers produced by the electrospinning process. In our work, we have, for the first time, determined the relationship between electrical parameters (electrical voltage, frequency of the pulses, and pulse duration) and the morphology of fiber mats obtained by the pulsed electrospinning of an alcoholic polyvinylpyrrolidone (PVP) solution. In addition, it was determined how the data obtained from the model correlate with those obtained experimentally.

## 2. Materials and Methods

### 2.1. Materials

Polyvinylpyrrolidone (PVP, $M_{w}$ = 1300 kDa) was purchased from Sigma-Aldrich. The PVP solution (17% concentration) was prepared by dissolving the polymer in 96% (*v*/*v*) pure ethanol, purchased from Polmos (Bielsko-Biala, Poland). The solution viscosity at 25 °C was 1.047 Pa·s.

### 2.2. Electrospinning Setup

**Figure 1:**
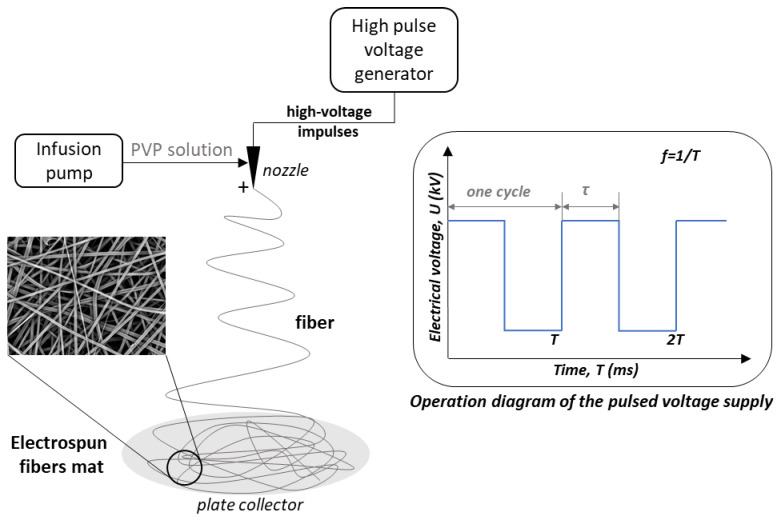
Scheme of the electrospinning process setup with an operational diagram of the pulsed voltage (PV) supply (blue line).

The electrospinning process was carried out using the setup shown in Figure 1. The system consisted of a high-voltage pulse generator, an infusion pump connected by a drain to a steel nozzle (inner/outer diameter = 0.63/0.9 mm), and a grounded circular aluminum collector (thickness = 0.12 mm and diameter = 100 mm) placed at a distance of 15 cm from the nozzle tip. The polymer solution was delivered to the nozzle at a flow rate of 0.9 mL/h. The electrospinning process was carried out using varying values of the electric voltage (*U*), the frequency of the pulses (*f*), and the pulse duration (*τ*). Electrical voltages of 8 kV and 15 kV were used. Electrical pulses were delivered with a frequency in the range of 10–100 Hz (changing in 10 Hz increments) and a duration in the range of 1–9 ms (changing in 1 ms increments). The resulting product was deposited on the collector for 3 min. A total of 180 experiments were carried out and as many different nonwovens were collected. All experiments were conducted at 25 °C. The ambient humidity did not exceed 40%.

### 2.3. Morphology of Electrospun Mats

The morphology of the obtained polymer electrospun mats was characterized by two parameters: the size of the average fiber diameter (*D*) and the uniformity of the fibers in terms of their diameter. Both parameters were determined based on images obtained by scanning electron microscopy (SEM, Hitachi TM-1000 (Hitachi High-Technologies Corporation, Tokyo, Japan)). Before the SEM images were taken, a thin layer of gold (about 10 nm) was sputtered onto each sample of the electrospun mats. Using the TM-1000 software (version 02-08) provided with the microscope, the diameters of 50 randomly selected fibers obtained by electrospinning the PVP solution under different electrical conditions of the process were measured. From the obtained values, the average diameters for each fiber (*D*), the standard deviation (±*SD*), and the coefficient of variation (*CV*) were calculated. The *CV* was calculated based on the following formula: $CV=\frac{SD}{D}\cdot 100\%.$

### 2.4. Factorial Design

The direct influence of process factors and the possible effects of their interaction can be predicted using the factor design method. The effects of selected electrical parameters of the electrospinning process (such as the electrical voltage (*U*), the frequency of the pulses (*f*), and the pulse duration (*τ*)) on the formation and morphology of the produced fibers were investigated using a factorial design.

In this method, in order to set up a two-level factor plan, it is necessary to define the experimental domain. Each factor is assigned a high (+) and low (−) level. The complete model system includes all combinations of extreme settings of experimental factors. A model with *k* factors consists of 2*^k^* experimental runs. For the case of three experimental factors, the response surface model is as follows:

(1) $$y=a_{0}+\sum_{i=1}^{3}a_{i}x_{i}+\sum_{i=1}^{3}\sum_{j=1}^{3}a_{ij}x_{i}x_{j}+a_{123}x_{1}x_{2}x_{3}$$

where *y* is the response; *a*_0_, *a_i_*, *a_ij_*, and *a*_123_ are the coefficients; and *x*_1_, *x*_2_, *x*_3_, *x_i_*, and *x_j_* are the experimental factors. The constant term *a*_0_ corresponds to the response value when all parameters are at the center point at an average level (*x*_1_ = *x*_2_ = *x*_3_ = 0).

The coefficients (*a*) are determined by the coded values of the factors (a high factor level is +1, and a low factor level is −1). For three factors, the experimental system takes the form of a matrix:

(2) $$\begin{array}{c} Experiment\;\;\;\;\;\;\;\;\;\\ 1\\ 2\\ 3\\ 4\\ 5\\ 6\\ 7\\ 8 \end{array}\left[\begin{array}{ccc} x_{1} & x_{2} & x_{3}\\ -1 & -1 & -1\\ -1 & -1 & +1\\ -1 & +1 & -1\\ -1 & +1 & +1\\ +1 & -1 & -1\\ +1 & -1 & +1\\ +1 & +1 & -1\\ +1 & +1 & +1 \end{array}\right]$$

To calculate the *a_i_*–*a_ijk_* coefficients, the X matrix is expanded with column *I* for the constant expression, and the columns for all possible factor interactions in the model are as follows:

(3) $$\begin{array}{c} Experiment\;\;\;\;\;\;\;\;\;\\ 1\\ 2\\ 3\\ 4\\ 5\\ 6\\ 7\\ 8 \end{array}\left[\begin{array}{cccccccc} I & x_{1} & x_{2} & x_{3} & x_{1}\cdot x_{2} & x_{1}\cdot x_{3} & x_{2}\cdot x_{3} & x_{1}\cdot x_{2}\cdot x_{3}\\ +1 & -1 & -1 & -1 & +1 & +1 & +1 & -1\\ +1 & -1 & -1 & +1 & +1 & -1 & -1 & +1\\ +1 & -1 & +1 & -1 & -1 & +1 & -1 & +1\\ +1 & -1 & +1 & +1 & -1 & -1 & +1 & -1\\ +1 & +1 & -1 & -1 & -1 & -1 & +1 & +1\\ +1 & +1 & -1 & +1 & -1 & +1 & -1 & -1\\ +1 & +1 & +1 & -1 & +1 & -1 & -1 & -1\\ +1 & +1 & +1 & +1 & +1 & +1 & +1 & +1 \end{array}\right]$$

Consequently, the experimental series can be summarized by means of the following matrix relation:

(4) y=X·A

which, in the described case, corresponds to following:

(5) $$\left[\begin{array}{c} y_{1}\\ y_{2}\\ y_{3}\\ y_{4}\\ y_{5}\\ y_{6}\\ y_{7}\\ y_{8} \end{array}\right]=\left[\begin{array}{cccccccc} +1 & -1 & -1 & -1 & +1 & +1 & +1 & -1\\ +1 & -1 & -1 & +1 & +1 & -1 & -1 & +1\\ +1 & -1 & +1 & -1 & -1 & +1 & -1 & +1\\ +1 & -1 & +1 & +1 & -1 & -1 & +1 & -1\\ +1 & +1 & -1 & -1 & -1 & -1 & +1 & +1\\ +1 & +1 & -1 & +1 & -1 & +1 & -1 & -1\\ +1 & +1 & +1 & -1 & +1 & -1 & -1 & -1\\ +1 & +1 & +1 & +1 & +1 & +1 & +1 & +1 \end{array}\right]\cdot \left[\begin{array}{c} a_{0}\\ a_{1}\\ a_{2}\\ a_{3}\\ a_{12}\\ a_{13}\\ a_{23}\\ a_{123} \end{array}\right]$$

And finally, the coefficients are determined by solving Equation (6) using the least squares method:

(6) $$A={\left(X^{T}\cdot X\right)}^{-1}\cdot X^{T}\cdot y$$

where *A* is the set of the coefficients, *X^T^* is the transposed matrix, and *y* is the response.

The absolute value of a given coefficient determines the influence of the analyzed model factor on the response. It is assumed that the higher the value of the coefficient, the stronger the relationship between the given factor and the response. In turn, the nature of this relationship is indicated by the sign of the coefficient: a positive coefficient means that the value of the response increases as the value of the factor increases, while a negative coefficient means that the relationship is inversely proportional.

### 2.5. The Selection of Process Factors for the Factorial Design

Conducting a 2^3^ full factorial analysis requires the selection of experimental variants for an electrospinning process carried out under extreme conditions. The process parameters are shown in Table 1. The high (maximum) and low (minimum) levels of process factors (*U*, *f*, and *τ*) were assigned values of +1 and −1, respectively. A frequency of the pulses of 30 Hz was chosen as the low frequency level, as below this frequency value, fiber electrodeposition does not occur.

### 2.6. Statistical Analysis

The PVP fiber diameter distributions are illustrated in the form of box-and-whisker plots prepared using the OriginPro 2023 software. 

Statistical differences between mean fiber diameters were determined using a one-way analysis of variance (ANOVA). The significance level (α) was set at 0.05, and data probabilities were considered statistically significant for *p* values < 0.05. The results are indicated in the graphs (Figures 5 and 7): (*) *p* < 0.05, (**) *p* < 0.01, and (***) *p* < 0.001.

## 3. Results and Discussion

### 3.1. Structure of Electrospun Mats

**Figure 2:**
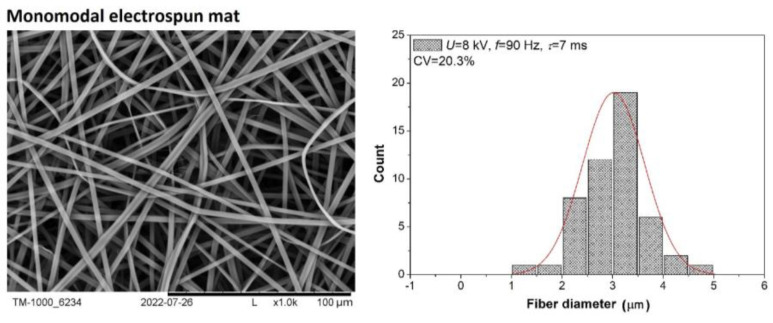
Morphology and distribution of fiber diameters in the electrospun mats produced at electrical voltage (*U*) = 8 kV, frequency of the pulses (*f*) = 90 Hz and pulse duration (*τ*) = 7 ms. Normal distribution (solid line on the graph).

**Figure 3:**
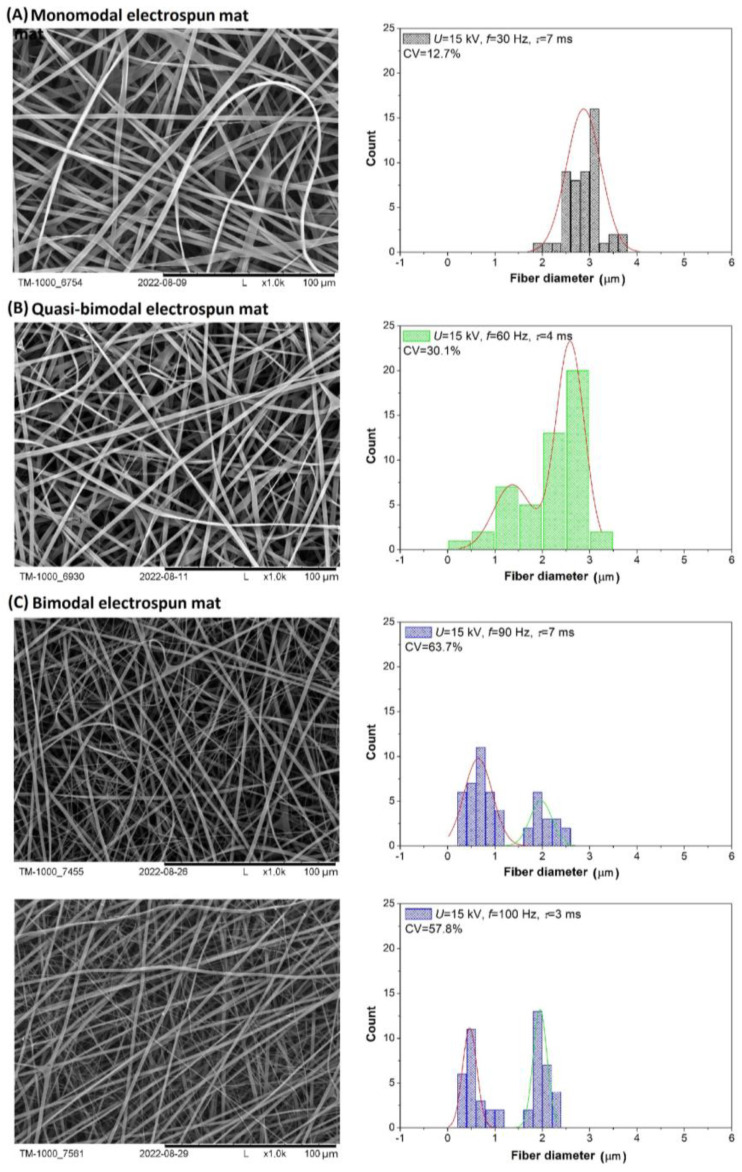
Morphology and distribution of fiber diameters in electrospun mats at electrical voltage *U* = 15 kV and at different values of the frequency of the pulse (*f*) and pulse duration (τ). Types of obtained nonwovens: monomodal (**A**), quasi-bimodal (**B**), and bimodal (**C**). Normal distribution (solid line on the graphs).

Figure 2 and Figure 3 show the SEM images of selected electrospun fiber mats obtained under specific process conditions, histograms, and estimated values of the coefficients of variation (CV %). The SEM images and histograms of the fiber diameter distribution were taken for nonwovens obtained during the electrospinning process carried out at electrical voltages of 8 kV or 15 kV, pulse frequencies in the range of 30–100 Hz, and pulse durations in the range of 1–9 ms. The examples presented (Figure 2 and Figure 3) show nonwovens with different fiber diameter distributions. Taking into account the histograms, three basic types of nonwovens were distinguished: monomodal (Figure 2 and Figure 3A), characterized by a low CV value and a single pick in the histogram; quasi-bimodal (Figure 3B), characterized by a CV value of 30% and 50% and two combined peaks per histogram CV; and bimodal (Figure 3C), with a high CV value and two well-separated peaks in the histogram.

In all examined cases, mats with smooth fibers were produced, and differences were only observed in the diameters of these fibers. When the system was operated at an electrical voltage of 8 kV and over the entire range of *f* (30–100 Hz) and *τ* (1–9 ms), mainly electrospun mats with a monomodal distribution of the fiber diameters were obtained (Figure 2). The CV values estimated for a sample of each nonwoven mat obtained under the specified conditions do not exceed 20% in most cases. In contrast, when the system was operated at 15 kV, the application of different conditions of the electrospinning process (*f* = 30–100 Hz and *τ* = 1–9 ms) led to electrospun mats with a monomodal, quasi-bimodal, and bimodal distribution of the fiber diameters in the electrospun mats (Figure 3). The obtained SEM images and histograms confirm a clear tendency for a parallel fraction of much thinner fibers to form when pulse frequencies above 60 Hz are applied (quasi-bimodal and bimodal fibers form). In the case of nonwovens with a bimodal distribution of the fiber diameter, two clear fractions of fibers with diameters in the range of 0.25–1.25 µm and above 1.25 µm can be identified. It has also been estimated that for a bimodal distribution of mean diameters, the CV values are high and in the range of 40–65%. Table 2 illustrates the dependence of the type of nonwoven obtained on the conditions under which the electrospinning process was carried out. We can see that the electrical parameters (*f* and *τ*) strongly affect the morphology of the obtained nonwovens. In general, we can expect to obtain bi- and quasi-bimodal fibers at frequencies in the range of 50–70 Hz, but only at high pulse durations. On the other hand, with f in the range of 80–100 Hz and τ in the range of 2–9 ms, we obtained mainly bimodal fibers (Table 2). Hence, we can conclude that a very large amount of the charge delivered to the system results in the appearance of an additional fraction of fine fibers.

As mentioned earlier, in recent years, the preparation of nonwovens with a bimodal distribution of the fiber diameter (both by needle-free electrospinning and by single- and multi-needle methods) has been of interest to many researchers. Recent reports speak of submicrofibrous membranes with a bimodal distribution, developed by Quan et al. [17], which exhibit an excellent breaking strength and elastic modulus. A little earlier than this, Zhao et al. [20] designed structured low-resistance fiber filters made of bimodal fibers and demonstrated that cleanable nanofiber membranes are able to rapidly transfer moisture and effectively capture harmful PM2.5 particles. In turn, Mei et al. [21] constructed fiber membranes with a bimodal structure using conventional single-needle electrospinning. Such nonwoven membranes showed high filtration efficiency, a low pressure drop, and higher quality factors compared to monomodal nonwoven membranes [21]. Bimodal structures have broad prospects not only for filtration materials and fiber scaffolds but also for applications in biomedicine and other fields. Rad et al. [19] fabricated a porous PCL/zein/gum Arabic nanofiber scaffold with a bimodal diameter distribution. Such scaffolds exhibited high hydrophilic properties, a favorable porosity (approximately 80%), and adequate tensile strength, which determines their high potential for application in skin tissue engineering. In another study, Soliman et al. [22] constructed multiscale three-dimensional scaffolds with controlled bimodal decomposition, which offers significant improvements over conventional monomodal scaffolds in terms of both their mechanical and biological performance. Such a novel scaffold exhibited better stiffness and strength values compared to conventional scaffolds and also had a more open pore structure, which increased cell motility and survival.

### 3.2. Effects of the Variables of the Pulsed Electrospinning Process on the Size of Polymer Fibers

The main objective of the present work was to investigate the influence of the electrical parameters of the process, such as the electrical voltage (*U*), the frequency of the pulses (*f*), and the pulse duration (*τ*), on the morphology of the resulting electrospun mats. The variants of the experiments along with the obtained results (average fiber diameter, *D*) are shown in Table 3.

In order to determine the model coefficients, a design matrix was prepared, assigning +1 values to the high levels of process factors and −1 values to low ones (Table 4).

The relationship between the parameters of the electrospinning process and the average diameter of the formed fibers can be expressed using Equation (7):

(7) $$D=a_{0}+a_{U}\cdot U+a_{f}\cdot f+a_{\tau }\cdot \tau +a_{Uf}\cdot Uf+a_{U\tau }\cdot U\tau +a_{f\tau }\cdot f\tau +a_{Uf\tau }\cdot Uf\tau$$

where $a_{U}$-$a_{Uf\tau }$ are the model coefficients; U, f, τ are the process factors (electrical voltage, frequency of the pulses, and pulse duration); and D is the response (average fiber diameter; µm).

The model matrix X (Table 5) was then constructed by adding column *I* to the design matrix, corresponding to the constant term $a_{0}$ in Equation (7).

The determined values of the $a_{0}—a_{Uf\tau }$ parameters are shown in Table 6.

After inserting the values obtained into Equation (7), the model equation (Equation (8)) describing the relationship between the electrical parameters and the mean diameter of the fibers was obtained.

(8) D=2.08−0.363U+0.328f+0.295τ−0.340Uf−0.013Uτ−0.483fτ+0.075Ufτ

Based on the obtained model coefficients (Table 6), the electrical voltage (*U*), the frequency of the pulses (*f*), and the pulse duration (*τ*) were found to have comparable effects on the mean values of the fiber diameters (*D*). The positive coefficients $a_{f}$ and $a_{\tau }$ indicate that the variables *D*, *f*, and *τ* are directly proportional and that the mean value of the fiber diameter (*D*) increases with an increasing *f* and *τ*. In contrast, the negative coefficient $a_{U}$ confirms that the variables *D* and *U* are inversely proportional, with the mean value of the fiber diameter decreasing as U increases. Furthermore, of the other model coefficients collected in Table 6 ($a_{Uf}$, $a_{U\tau }$, $a_{f\tau }$, and $a_{Uf\tau }$), only the variable *D* and the product of *U*, *f*, and *τ* are also directly proportional, as confirmed by the positive coefficient $a_{Uf\tau }$. However, the change in the fiber diameter (*D*) is most affected by the product of *f* and *τ*, as evidenced by the highest value of 0.483 of the coefficient $a_{f\tau }$.

**Figure 4:**
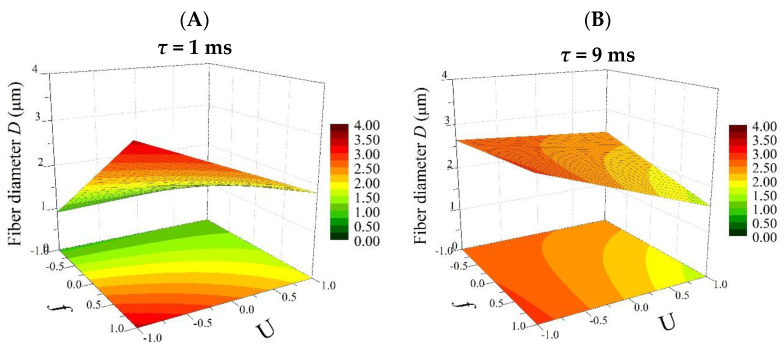
Response surface plots presenting the dependence of the diameter of the obtained fibers (*D*) on the frequency of the pulses (*f*) and the electrical voltage (*U*) for (**A**) the minimum pulse duration (*τ* = −1; 1 ms) and (**B**) the maximum pulse duration (*τ* = 1; 9 ms).

Based on Equation (8), 3D plots were generated (Figure 4, Figures 6 and 8), which allow for a comprehensive description of the interplay between the electrical parameters and the diameters of the obtained fibers.

Figure 4 shows the effect of the frequency (*f*) and electrical voltage (*U*) on the diameter of the fibers produced (*D*) at the minimum (1 ms) and maximum (9 ms) pulse durations (*τ*).

For pulse durations (*τ*) at a low level (1 ms) (Figure 4A), the largest changes in diameter were observed. An increase in the average fiber diameter (from 0.75 to even more than 3 µm) is directly proportional to an increasing value of the frequency in the process carried out. At the same time, the relationship between the average fiber diameter and the electrical voltage is inversely proportional (the higher the voltage, the thinner the fibers obtained). In addition, it is important to highlight the fact that with *τ* at a low level (1 ms), both electrical parameters (*f* and *U*) affect the size of the polymer fiber. Thus, the valuable information obtained from the factor analysis shows that the greatest possibility of controlling the electrospinning process to obtain the desired product is observed at *τ* = 1 ms. On the other hand, with *τ* at a high level (9 ms), the thickness of the fiber depends only on changes in the electrical voltage (*U*) (Figure 4B), but the range of changes in the diameter is incomparably smaller than for *τ* = 1 ms.

**Figure 5:**
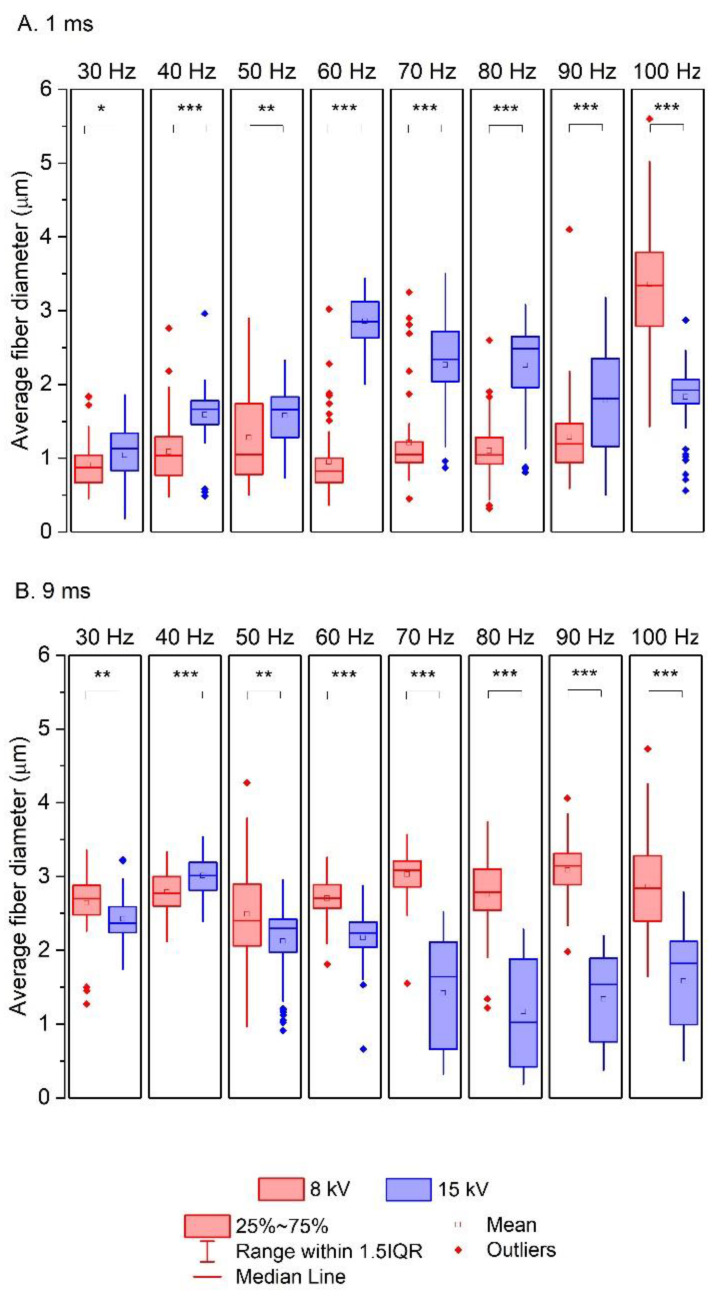
The effect of electrical voltage (*U*) on the average fiber diameters obtained by electrospinning depending on the pulse duration: (**A**) 1 ms and (**B**) 9 ms were used in the process; (*) *p* < 0.05, (**) *p* < 0.01, and (***) *p* < 0.001.

Similar effects were observed when analyzing the results obtained experimentally (Figure 5). The application of an electrical voltage of 15 kV and a pulse duration of 1 ms leads to thicker fibers (blue boxes; Figure 5A). On the other hand, when a pulse duration of 9 ms is used, the application of a high electrical voltage leads to thinner fibers than in a process with a lower electrical voltage (Figure 5B), especially when a frequency of the pulse of above 60 Hz is used. At lower *f* values (below 60 Hz), the average fiber diameters obtained at 8 kV and 15 kV are similar (Figure 5B). In addition, the statistical analysis carried out showed that the differences between the average fiber diameters of the experiments carried out are statistically significant (with a significance level of *p* < 0.001 in most cases (*** in Figure 5)). It is also worth noting that the blue boxes in the box-and-whisker plots (Figure 5B, 9 ms) are longer than the red boxes, indicating a greater variability in the mean fiber diameters obtained experimentally.

**Figure 6:**
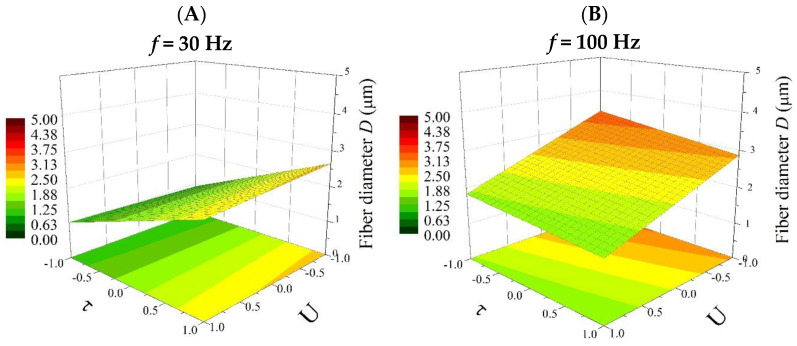
Response surface plots presenting the dependence of the diameter of the obtained fibers *(D)* on the pulse duration (*τ*) and the electrical voltage (*U*) for (**A**) the minimum frequency of the pulses (*f* = −1; 30 Hz) and (**B**) the maximum frequency of the pulses (*f* = 1; 100 Hz).

Figure 6 shows the effects of the pulse duration (*τ*) and the electrical voltage (*U*) on the diameter of the produced fibers (*D*) at the minimum (30 Hz) and maximum (100 Hz) frequencies (*f*).

For frequencies at low levels (30 Hz) (Figure 6A), the pulse duration (*τ*) affects the diameter of the fibers obtained to a much greater extent than the electrical voltage (*U*), and changing *τ* from low to high values (from 1 ms to 9 ms) increases *D* from approximately 0.75 µm to 1.75 µm. Furthermore, changing *U* from a low value to a high value (from 8 kV to 15 kV) causes only a small change in the thickness of the obtained fibers (from 1.75 to 2.0 µm). The situation is the opposite for the maximum frequency (100 Hz) (Figure 6B). The electrical voltage then affects the diameters of the resulting fibers, and the *D* values change: in the case of an 8 kV electrical operation, the average fiber diameters are above 2.0 µm, while in the case of a 15 kV electrical operation, the *D* values are equal to approximately 1.25 µm. In contrast, the pulse duration (*τ*) in this case has no effect on the thickness of the produced fibers.

**Figure 7:**
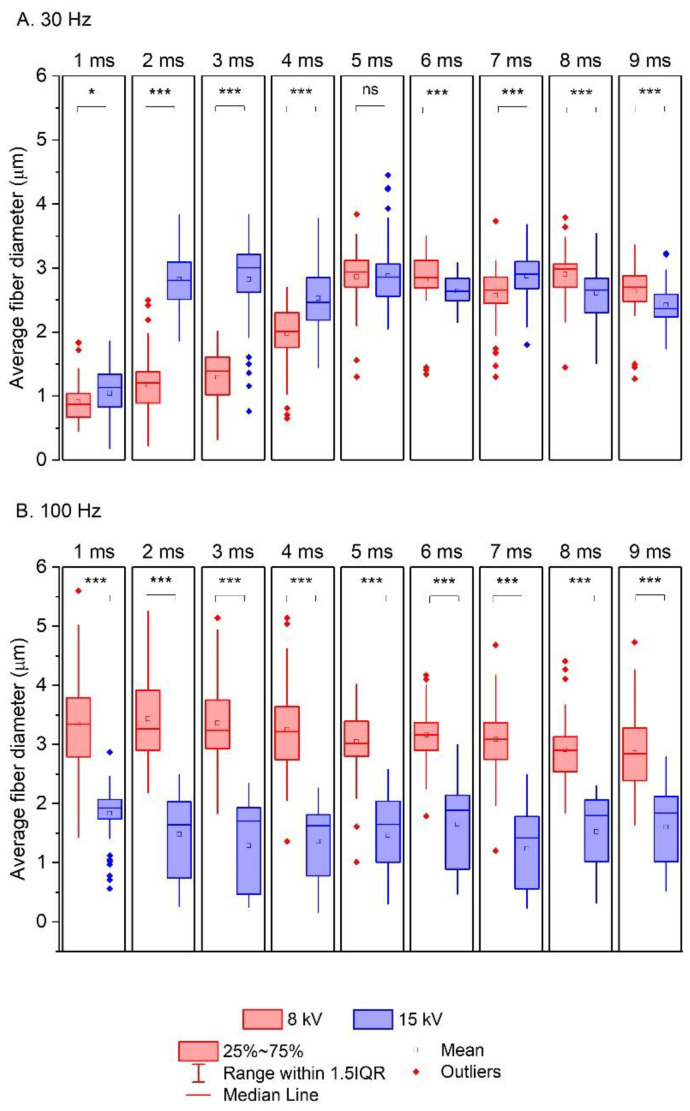
The effect of the electrical voltage (*U*) on the average fiber diameters obtained by electrospinning depending on the frequency of the pulses: (**A**) 30 Hz and (**B**) 100 Hz were used in the electrospinning process; (*) *p* < 0.05, and (***) *p* < 0.001.

The results obtained under the 2^3^ full factorial design correspond very well to those obtained experimentally (Figure 7). When using a frequency of 30 Hz and a pulse duration in the range of 5–9 ms, the effect of the electrical voltage (*U*) on the average fiber diameter (*D*) is negligible (Figure 7A). Differences in the fiber thickness produced at 8 and 15 kV are only apparent when *τ* = 2–4 ms. On the other hand, for *f* = 100 Hz, the fibers obtained at a high electrical voltage (15 kV) are half as thin as those obtained at a lower electrical voltage (8 kV) (Figure 7B). Furthermore, the differences between the average fiber diameters of the experiments are statistically significant (except for the fibers produced at *f* = 30 Hz and *τ* = 4 ms, Figure 7A). As mentioned earlier, a longer box indicates a greater variability in the mean fiber diameters, while a shorter box indicates less variability. Thus, the longer blue boxes (Figure 7B) are associated with a bimodal and quasi-bimodal distribution of the mean fiber diameters of electrospun fibers at 15 kV (see Table 2).

**Figure 8:**
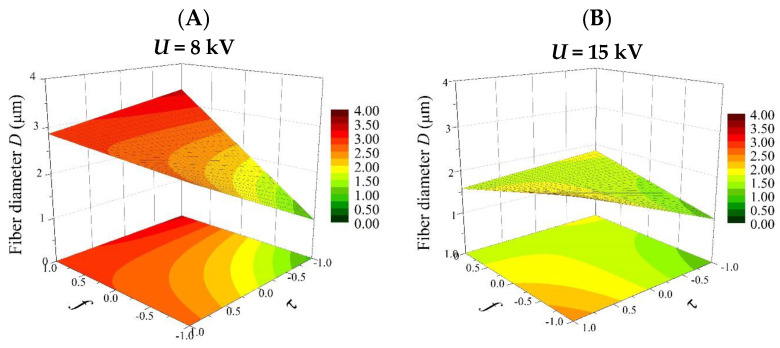
Response surface plots presenting the dependence of the diameter of the obtained fibers *(D)* on the frequency of the pulses (*f*) and the pulse duration (*τ*) for (**A**) the minimum electrical voltage (*U* = −1; 8 kV) and (**B**) the maximum electrical voltage (*U* = 1; 15 kV).

Figure 8 shows the effects of the frequency (*f*) and pulse duration (*τ*) on the diameter of the fibers produced (*D*) at the minimum (8 kV) and maximum (15 kV) electrical voltage (*U*).

For an electrical voltage (*U*) at a low level (8 kV) (Figure 8A), the pulse duration (*τ*) affects the fiber diameter much more than the frequency (*f*). A change in f (from 30 Hz to 100 Hz) causes little change in the value of *D* (2.5–2.72 µm). However, a change in *τ* (from 1 ms to 9 ms) causes the diameter to increase gradually from low values (about 1 µm) to high values (2.75 µm). This confirms that by operating only on the pulse duration, fibers of a desired thickness can be easily obtained. Interestingly, for an electrical voltage at a high level (15 kV), the fiber diameters decrease (from 2.25 to 1.5 µm) with an increasing frequency (*f*) and increase (from 1 to 2 µm) with an increasing pulse duration (Figure 8B). In this case, there is also a specific range of coefficients (the light-green area in Figure 8B), in which even small changes in their values do not result in changes in the value of the average fiber diameter.

**Figure 9:**
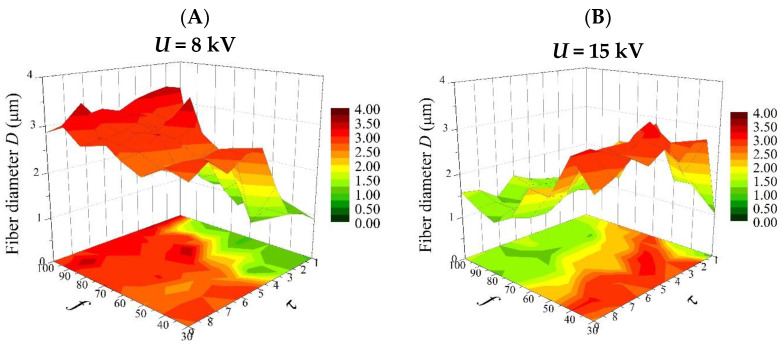
Dependence of average fiber diameters (*D*) on the frequency of the pulses (30–100 Hz) and pulse duration (1–9 ms) for a process conducted at (**A**) *U* = 8 kV and (**B**) 15 kV.

From the perspective of the practical use of a factor analysis, it is important to compare the relationships obtained with the model with those obtained from experiments. Figure 9 shows the relationship between a frequency of the pulses (*f*) in the range 30–100 Hz, a pulse duration (*τ*) in the range 1–9 ms, and the average diameter of the fibers produced (*D*) at electrical voltages (*U*) of 8 kV and 15 kV. The graphs were produced using only experimentally obtained data. For an electrical voltage of 8 kV, varying the pulse duration in the range of 1–9 ms leads to fibers with diameters in the range of 0.9–3.0 µm, while for higher pulse durations (5–9 ms) and over the entire set frequency range (30–100 Hz), the average diameters oscillate between 2.5 and 3.0 µm (Figure 9A). In addition, it is clear that the mean diameters of the fibers obtained by electrospinning depend much less on the frequency than on the pulse duration. These results correspond very well with those obtained from the factor analysis (Figure 8A). In both cases, we observe a clear dependence of *D* on *τ*. In the case of an electrical voltage of 15 kV, an inverse relationship is observed: the mean diameters of the fibers obtained by electrospinning depend more on the frequency used in the experiments (Figure 9B). When frequencies in the range of 30–60 Hz and pulse durations in the range of 2–9 ms were used, fibers with thicknesses oscillating between 2.5 and 3.0 µm were obtained. Thinner fibers were obtained only when *τ* = 1 ms was used. Furthermore, for *f* = 75–100 Hz and *τ* = 2–9 ms, fibers in the range of 1–2 µm were obtained. This reduction in the average fiber diameter is related to the appearance, at a high *f*, of an additional fine fiber fraction, as described in the previous section. In order to examine the correctness of fit of the model to the experimental data, the model was verified using statistical methods. R-squared (coefficient of determination) goodness-of-fit summary statistics were performed. Two data arrays were analyzed: experimental data and theoretical data, calculated using the formula describing the model. The function used returns the Square of the Pearson Product–Moment Correlation Coefficient between two arrays of data. When comparing the experimental results of the diameters with the calculated theoretical values (Equation (8)) obtained for an electrical voltage of 8 kV, the coefficient of determination was equal to 48 percent, which corresponds to a correlation coefficient, “r”, of 0.69. The strength of correlation in the range of 0.5–0.7 for the classification, according to J. Guilford, is a high correlation, confirming a good model fit. The coefficient of determination, calculated for the sets of experimental and theoretical diameter values obtained at an electrical voltage of 15 kV, is equal to 1 percent. The correlation coefficient, “r”, in this case, is 0.1, which is a very low model correlation for the classification, according to J. Guilford. The low value of the correlation coefficient, “r”, may be due to the appearance of a fraction of fine fibers (quasi-bimodal and bimodal nonwovens), which the computational model does not take into account in any way. The appearance of an additional fraction of fine fibers explains the poor fit of the 3D surface plots created from the model data (Figure 8B) and the experimental data (Figure 9B).

As mentioned earlier, when an electrical voltage of 15 kV is applied, a large scatter in the average diameters of the obtained fibers is observed. Therefore, in this work, a factor analysis was also applied to determine the effects of the electrical parameters of the process, and in this case, only the pulse frequency (*f*) and pulse duration (*τ*), on the standard deviation estimated for the average fiber diameters obtained from the electrospinning process, were considered. The experimental variants, together with the obtained results (standard deviation, *SD*) are shown in Table 7. We decided that the *f* range (40–70 Hz) was the most authoritative due to the occurrence of all structures (mono-, quasi-, and bimodal) under these conditions.

The effects of the electrospinning process parameters (in this case, only *f* and *τ*) on the standard deviation (*SD*) for the mean fiber diameters can be expressed by Equation (9):

(9) $$SD=a_{0}+a_{f}\cdot f+a_{\tau }\cdot \tau +a_{f\tau }\cdot f\tau$$

where $a_{f}$, $a_{\tau }$, $a_{f\tau }$ are the model coefficients; f, τ are the process factors (the frequency of the pulses and pulse duration); and SD is the response (standard deviation).

To determine the model coefficients as described above, a design matrix was prepared, assigning the high levels of process factors a value of +1 and the low levels, a value of −1 (Table S1).

The model matrix X (Table S2) was then constructed by adding column *I* to the design matrix, corresponding to the constant term $a_{0}$ in Equation (9).

The determined values of the $a_{0}-a_{f\tau }$ parameters are shown in Table 8.

After inserting the values obtained into Equation (9), the model equation (Equation (10)) describes the relationship between the electrical parameters and the standard deviation.

(10) SD=0.50+0.16f+0.01τ+0.08fτ

Based on the obtained model coefficients (Table 8), it was found that the standard deviation (*SD*) of the mean values of the obtained fiber diameters is most influenced by the frequency of the pulses (*f*). Of much lesser importance is the pulse duration (*τ*).

## 4. Conclusions

In this study, an attempt was made, for the first time, to determine how the electrical parameters of the pulsed electrospinning process, namely, the electrical voltage (*U*), the frequency of the pulses (*f*), and the pulse duration (*τ*), affect the morphology of manufactured nonwovens. It was found that by appropriately selecting the electrical process parameters (*U*, *f*, and *τ*), the thickness of the obtained fibers can be controlled, and, consequently, nonwovens with a desired morphology can be obtained.

A detailed description of the relationships of the electrical parameters of the electrospinning process, and the structure of the resulting mats, was made possible by using a 2^3^ factorial design. It was shown that the positive coefficients $a_{f}$ (0.328) and $a_{\tau }$ (0.295) indicate that the variables *D*, *f*, and *τ* are directly proportional and that the mean value of the fiber diameter (*D*) increases with an increasing *f* and *τ*. In contrast, the negative coefficient $a_{U}$ (−0.363) confirms that the variables *D* and *U* are inversely proportional, with the mean value of the fiber diameter decreasing as *U* increases.

In addition, in order to check the validity of the model’s fit to the experimental data, it was verified using statistical methods. It was shown that the correlation coefficient, “r”, for the data obtained under an 8 kV electrical voltage is 0.69, while for the data under 15 kV, it is 0.1. The strength of the correlation in the range of 0.5–0.7, according to J. Guilford’s classification, is a high correlation, confirming a good model fit. In contrast, the very low model correlation for the data under 15 kV is explained by the appearance of an additional fraction of fine fibers, which the computational model does not take into account in any way. Thus, it was confirmed that it is not necessary to carry out multiple tests to find a nonwoven with a suitable morphology and that the factor analysis proves itself as a useful tool for the design of electrospinning processes.

Furthermore, it was found that the electrospinning process carried out at an electrical voltage of 8 kV leads to nonwovens with a monomodal fiber distribution. Under these conditions and using lower pulse durations (*τ* < 5 ms), it was possible to obtain fibers with different diameters (from 1 to more than 3 µm), while using higher pulse durations (*τ* > 5 ms) produced coarse fibers (*D* = approx. 3 µm). The electrospinning process carried out under an electrical voltage of 15 kV, with frequency of the pulses (*f*) in the range of 30–50 Hz and pulse durations (*τ*) above 2 ms, leads to fibers with average diameters of approx. 2.5–3 µm. In contrast, when *f* = 80–100 Hz, the average diameters of the obtained fibers are about 1.5 µm. In addition, the application of an electrical voltage of 15 kV and with a high frequency of the pulse (80–100 Hz), we observe the coexistence of a fraction of finer and thicker fibers. According to the available literature, such nonwovens with a bimodal fiber distribution may have a number of interesting applications.

## Figures and Tables

**Table 1:** Process variables selected for the 2^3^ factorial analysis for the fiber diameter in fibrous mats.

Parameter	Low Level (−1)	High Level (+1)
*U*—electrical voltage	8 kV	15 kV
*f*—frequency of the pulses	30 Hz	100 Hz
*τ*—pulse duration	1 ms	9 ms

**Table 2:** Process conditions for obtaining monomodal, quasi-bimodal, and bimodal electrospun mats at *U* = 15 kV.

15 kV		Pulse Duration [ms]								
		1	2	3	4	5	6	7	8	9
Frequency of the pulses (Hz)	30									
	40									
	50									
	60									
	70									
	80									
	90									
	100									
	Monomodal		Quasi-bimodal					Bimodal		

**Table 3:** Experimental variants for a 2^3^ factorial design, including the average diameter of the obtained fibers (*D*). SD is the standard deviation.

No.	*U* (kV)	*f* (Hz)	*τ* (ms)	*D* (µm)	±*SD* (µm)
1	8	30	1	0.91	0.31
2	8	30	9	2.64	0.44
3	8	100	1	3.36	0.88
4	8	100	9	2.86	0.66
5	15	30	1	1.04	0.40
6	15	30	9	2.42	0.30
7	15	100	1	1.83	0.46
8	15	100	9	1.58	0.66

**Table 4:** Design matrix of a 2^3^ factorial design.

No.	*U*	*f*	*τ*
1	−1	−1	−1
2	−1	−1	+1
3	−1	+1	−1
4	−1	+1	+1
5	+1	−1	−1
6	+1	−1	+1
7	+1	+1	−1
8	+1	+1	+1

**Table 5:** Model matrix of a 2^3^ factorial design.

No.	*I*	U	f	τ	Uf	Uτ	fτ	Ufτ
1	+1	−1	−1	−1	+1	+1	+1	−1
2	+1	−1	−1	+1	+1	−1	−1	+1
3	+1	−1	+1	−1	−1	+1	−1	+1
4	+1	−1	+1	+1	−1	−1	+1	−1
5	+1	+1	−1	−1	−1	−1	+1	+1
6	+1	+1	−1	+1	−1	+1	−1	−1
7	+1	+1	+1	−1	+1	−1	−1	−1
8	+1	+1	+1	+1	+1	+1	+1	+1

**Table 6:** The model coefficients determining the impact of electrical voltage (U), frequency of the pulses (f), and pulse duration (τ) on the average fiber diameter (D).

$a_{0}$	$a_{U}$	$a_{f}$	$a_{\tau }$	$a_{Uf}$	$a_{U\tau }$	$a_{f\tau }$	$a_{Uf\tau }$
2.08	−0.363	0.328	0.295	−0.340	−0.013	−0.483	0.075

**Table 7:** Experimental variants for a 2^2^ factorial design with the obtained mean standard deviation (*SD*).

No.	*U* (kV)	*f* (Hz)	*τ* (ms)	*D* (µm)	±*SD* (µm)
5	15	40	1	1.59	0.41
6	15	40	9	3.01	0.26
7	15	70	1	2.26	0.57
8	15	70	9	1.42	0.74

**Table 8:** The model coefficients determining the impact of the frequency of the pulses (f) and pulse duration (τ) on the standard deviation (SD).

$a_{0}$	$a_{f}$	$a_{\tau }$	$a_{f\tau }$
0.50	0.16	0.01	0.08

## Data Availability

The original contributions presented in the study are included in the article/Supplementary Material, further inquiries can be directed to the corresponding author.

## Supplementary Materials

The following supporting information can be downloaded at: https://www.mdpi.com/article/10.3390/polym16162352/s1, Table S1: Design matrix of a 2^2^ factorial design; Table S2: Model matrix of a 2^2^ factorial design
