# Supplementary material for: The Effects of Pulsed Electrospinning Process Variables on the Size of Polymer Fibers Established with a 23 Factorial Design
Source: Polymers (Basel). 2024 Aug 20;16(16):2352. doi: 10.3390/polym16162352 (PMC11360675; doi:10.3390/polym16162352)
Supplement: Supplementary file 1 [file polymers-16-02352-s001.zip › polymers-3079327-supplementary.pdf]

SUPPLEMENTARY MATERIAL

Table S1. Design matrix of a 2^2 factorial design

No.	f	τ
1	-1	-1
2	-1	$+1$
3	$+1$	-1
4	$+1$	$+1$

Table S2. Model matrix of a 2^2 factorial design

No.	I	f	τ	$f\tau$
1	$+1$	-1	-1	$+1$
2	$+1$	-1	$+1$	-1
3	$+1$	$+1$	-1	-1
4	$+1$	$+1$	$+1$	$+1$
